# Establishment of a Novel Combined Nomogram for Predicting the Risk of Progression Related to Castration Resistance in Patients With Prostate Cancer

**DOI:** 10.3389/fgene.2022.823716

**Published:** 2022-05-10

**Authors:** Shuqiang Li, Lei Shi, Fan Li, Bing Yao, Liansheng Chang, Hongyan Lu, Dongkui Song

**Affiliations:** ^1^ Department of Urology, The First Affiliated Hospital of Zhengzhou University, Zhengzhou, China; ^2^ Department of Urology, The Third Affiliated Hospital of Chongqing Medical University, Chongqing, China

**Keywords:** prostate cancer, castration resistance, progression, nomogram, KIFC2, BCAS1

## Abstract

**Background:** The emergence of castration resistance is fatal for patients with prostate cancer (PCa); however, there is still a lack of effective means to detect the early progression. In this study, a novel combined nomogram was established to predict the risk of progression related to castration resistance.

**Methods:** The castration-resistant prostate cancer (CRPC)-related differentially expressed genes (DEGs) were identified by R packages “limma” and “WGCNA” in GSE35988-GPL6480 and GSE70768-GPL10558, respectively. Relationships between DEGs and progression-free interval (PFI) were analyzed using the Kaplan–Meier method in TCGA PCa patients. A multigene signature was built by lasso-penalized Cox regression analysis, and assessed by the receiver operator characteristic (ROC) curve and Kaplan–Meier curve. Finally, the univariate and multivariate Cox regression analyses were used to establish a combined nomogram. The prognostic value of the nomogram was validated by concordance index (C-index), calibration plots, ROC curve, and decision curve analysis (DCA).

**Results:** 15 CRPC-related DEGs were identified finally, of which 13 genes were significantly associated with PFI and used as the candidate genes for modeling. A two-gene (KIFC2 and BCAS1) signature was built to predict the risk of progression. The ROC curve indicated that 5-year area under curve (AUC) in the training, testing, and whole TCGA dataset was 0.722, 0.739, and 0.731, respectively. Patients with high-risk scores were significantly associated with poorer PFI (*p* < 0.0001). A novel combined nomogram was successfully established for individualized prediction integrating with T stage, Gleason score, and risk score. While the 1-year, 3-year, and 5-year AUC were 0.76, 0.761, and 0.762, respectively, the good prognostic value of the nomogram was also validated by the C-index (0.734), calibration plots, and DCA.

**Conclusion:** The combined nomogram can be used to predict the individualized risk of progression related to castration resistance for PCa patients and has been preliminarily verified to have good predictive ability.

## Introduction

The global cancer statistics showed that more than 19 million people had been newly diagnosed with cancer around the world in 2020, and approximately 10 million cases died of cancer in the same period, indicating that cancer has now been a leading cause of death and a significant health burden in many countries globally (Sung et al., 2021). Furthermore, it was estimated that there will be 6.9 million new cancer cases only among the elderly over 80 years by 2050 with an increased rate of over 200% (Pilleron et al., 2021). For males, prostate cancer (PCa) is now the second most frequently occurring cancer worldwide, accounting for 14.1% (1.4 million cases) across all cancer types in 2020 (Sung et al., 2021). Concurrently, PCa is one of the leading causes of cancer-specific death in men, with the number of deaths exceeding 350,000 per year (Rebello et al., 2021). Although a variety of new diagnosis and treatment options have emerged in recent years, PCa will eventually progress to castration-resistant prostate cancer (CRPC) in almost all patients with advanced prostate cancer, resulting in great challenges in the therapeutic selection and a serious impact on the overall survival (Nuhn et al., 2019; Teo et al., 2019).

Once CRPC occurs, either non-metastatic CRPC (nmCRPC) or mCRPC, the survival benefit of patients receiving routine treatment will be significantly reduced because of the more progressive biological characteristics. The mechanisms of castration resistance in PCa are various, mainly including the changes in androgen receptor (AR) and non-AR signaling pathways (Feldman and Feldman, 2001; Maitland, 2021). Thanks to the experts’ efforts, the most effective therapeutic agents (abiraterone, enzalutamide, docetaxel, and radium-223) and novel agents (sipuleucel-T, lutetium-177, and olaparib) have all been confirmed by clinical practice and trials to have substantial benefits (de Bono et al., 2020; Gillessen et al., 2020; Sandhu et al., 2021). However, the dilemma for clinicians is still unsolvable when determining the optimal treatment sequencing and application timing of drugs. Therefore, it is urgent to find new markers that can accurately predict the castration resistance and distinguish CRPC from PCa earlier to guide the clinical treatment schedules in real time.

With the rapid development of genomics, a large number of genomic data were obtained from the microarray chip and high-throughput sequencing, which helped us to better understand the tumor heterogeneity and guide future clinical decision-making (Miyamoto et al., 2018; Labrecque et al., 2019; Puhr et al., 2021; Ylitalo et al., 2021). For example, the SPP1 gene was identified as an extracellular matrix signature, which was remarkably up-regulated in mCRPC and may be a novel therapeutic target for mCRPC patients (Pang et al., 2019). Based on the transcriptome profiles, a CRPC-derived prognosis signature was developed to predict the recurrence-free survival, overall survival, and metastasis-free survival in PCa patients (A et al., 2021). However, markers currently available to predict the progression related to castration resistance in PCa patients are still lacking.

In this study, we first downloaded the transcriptome data and clinical information from the public genome databases, and then comprehensively analyzed the gene expression matrix and its relationship with clinical characteristics using R packages. Finally, we established a novel combined nomogram to predict the individualized risk of progression related to castration resistance and may be helpful in the planning of therapeutic strategies. The workflow diagram is shown in Supplementary Figure S1.

## Materials and Methods

### Data Collection and Preparation

The gene expression profiling data in GSE35988 based on platform GPL6480 (GSE35988–GPL6480), and GPL6848 (GSE35988–GPL6848) and GSE70768 based on platform GPL10558 (GSE35988-GPL10558) were downloaded from the Gene Expression Omnibus (GEO) (https://www.ncbi.nlm.nih.gov/geo/), each of which contains benign prostate tissue (normal), PCa, and CRPC samples. The gene expression profile by RNAseq and clinical information of PCa patients in the Cancer Genome Atlas (TCGA) were obtained from the online platform of UCSC Xena (Goldman et al., 2020). All the gene expression data from GEO and UCSC Xena were log_2_ transformed. The missing value was supplemented by the k-nearest neighbor method using R package “impute.”

### Identification of CRPC-Related Differentially Expressed Genes (CRPC-DEGs)

With the cutoff criteria of adjusted *p*-value < 0.05 and log_2_ fold change |FC| > 1, the DEGs of CRPC compared with normal prostate and PCa samples were identified by R package “limma” in GSE35988 and GSE70768, respectively. Then, the Venn analysis was performed by a webtool jvenn (http://jvenn.toulouse.inra.fr/app/example.html) to obtain the CRPC-DEGs distinguishing CRPC from normal and PCa samples in both GSE35988 and GSE70768. The heatmap and volcano plots were performed by R package “pheatmap” and “ggplot2” to visualize the DEGs.

### Weighted Gene Co-Expression Network Analysis

The WGCNA was performed using the gene expression matrix of GSE35988–GPL6480 and GSE70768–GPL10558, respectively, by R package “WGCNA” (Langfelder and Horvath, 2008). First, sample clustering was constructed to detect the outliers. Then, the optimal soft threshold power (β) was selected with the R-square = 0.85 for the transformation from the gene expression matrix to the topological overlap matrix (TOM). Based on the dissimilarity measure of TOM, the dynamic tree cutting method was used to divide genes into different modules, and the Eigengene adjacency heatmap was performed to show the gene expression in each module. The correlation between modules and clinical traits was analyzed using Pearson’s correlation test. Finally, genes in the CRPC correlated modules were selected for further analysis.

### Functional Enrichment Analysis and Construction of the Protein–Protein Interaction (PPI) Network

The genes in CRPC-correlated modules were used for functional enrichment analysis of Gene Ontology (GO) term and the Kyoto Encyclopedia of Genes and Genomes (KEGG) pathway. With a cutoff value of *p* < 0.05, the analysis and visualization of GO and KEGG were conducted on web tool “Metascape” (Zhou et al., 2019). The PPI network was constructed by Metascape, Cytoscape (Shannon et al., 2003), and the Search Tool for the Retrieval of Interacting Gene Database (STRING) (Szklarczyk et al., 2021).

### Construction and Evaluation of a Multigene Signature

To determine the final CRPC-DEGs, we first obtained the CRPC-associated genes (CRPC genes) in both GSE35988–GPL6480 and GSE70768–GPL10558 by Venn analysis of genes in CRPC-correlated modules (negative and positive), and then took their intersection with the CRPC-DEGs identified by R package “limma.” For the final CRPC-DEGs, Kaplan–Meier survival analysis was performed in the TCGA dataset to identify the progression-free interval (PFI) associated CRPC-DEGs as the candidate genes for building the multigene signature. Afterward, all the PCa patients in the whole TCGA dataset were separated into the training and testing group randomly by using the R package “caret.” Next, the ideal prognostic genes and their regression coefficients (β) were screened out by lasso-penalized Cox regression analysis when the optimal lambda value was identified by R package “glmnet.” The risk score of each PCa patient in the training group was calculated based on the following formula:

$$Risk\;score=\sum_{i=1}^{n}\beta i\times Expi.$$

Then, PCa patients in the training group were separated into the high-risk and low-risk groups using the optimal cutoff value, which was calculated by the “surv_cutpoint” function of R package “survminer.” The Kaplan–Meier survival analysis was performed using the R package “survival” to assess the difference in PFI between the high-risk and low-risk groups. Finally, the receiver operator characteristic (ROC) curve was used to evaluate the effectiveness of the multigene signatures in prognosis. The prognostic power of multigene signature was also assessed in the testing and whole dataset.

### Gene and Protein Expression Profiles of the Genes in the Model

The expression level of each gene in the prognostic model was analyzed by multiple comparisons of one-way ANOVA in different groups. The adjusted *p*-value < 0.05 was considered significant. The Human Protein Atlas (HPA) database was used to explore the protein expression levels of each gene in normal and tumor tissues.

### Establishment and Validation of a Nomogram Based on the Cox Analysis

The univariate and multivariate Cox regression models were used to analyze the relationship among the PFI and characteristics of risk score, age, T stage, N stage, laterality, Gleason score, and PSA by R packages “survival” and “survminer.” A predictive nomogram was established based on the results of multivariate Cox regression analysis. The concordance index (C-index) and the ROC curve were used to evaluate the discriminating ability of the nomogram. The calibration plots and decision curve analysis (DCA) were used to assess the predictive power and clinical utility of the nomogram.

### Statistical Analysis

CRPC-DEGs were identified using R package “limma.” The CRPC-related genes and modules were identified using R package “WGCNA.” Lasso-penalized Cox regression analysis was used to build the multigene signature, which was assessed using the Kaplan–Meier survival analysis and ROC curve. The nomogram was established using the univariate and multivariate Cox regression analyses, and validated by the C-index, ROC curve, calibration plots, and DCA. Data in this study were analyzed using R software with a *p*-value < 0.05 being considered statistically significant.

## Results

### Identification of CRPC-DEGs

**FIGURE 1:**
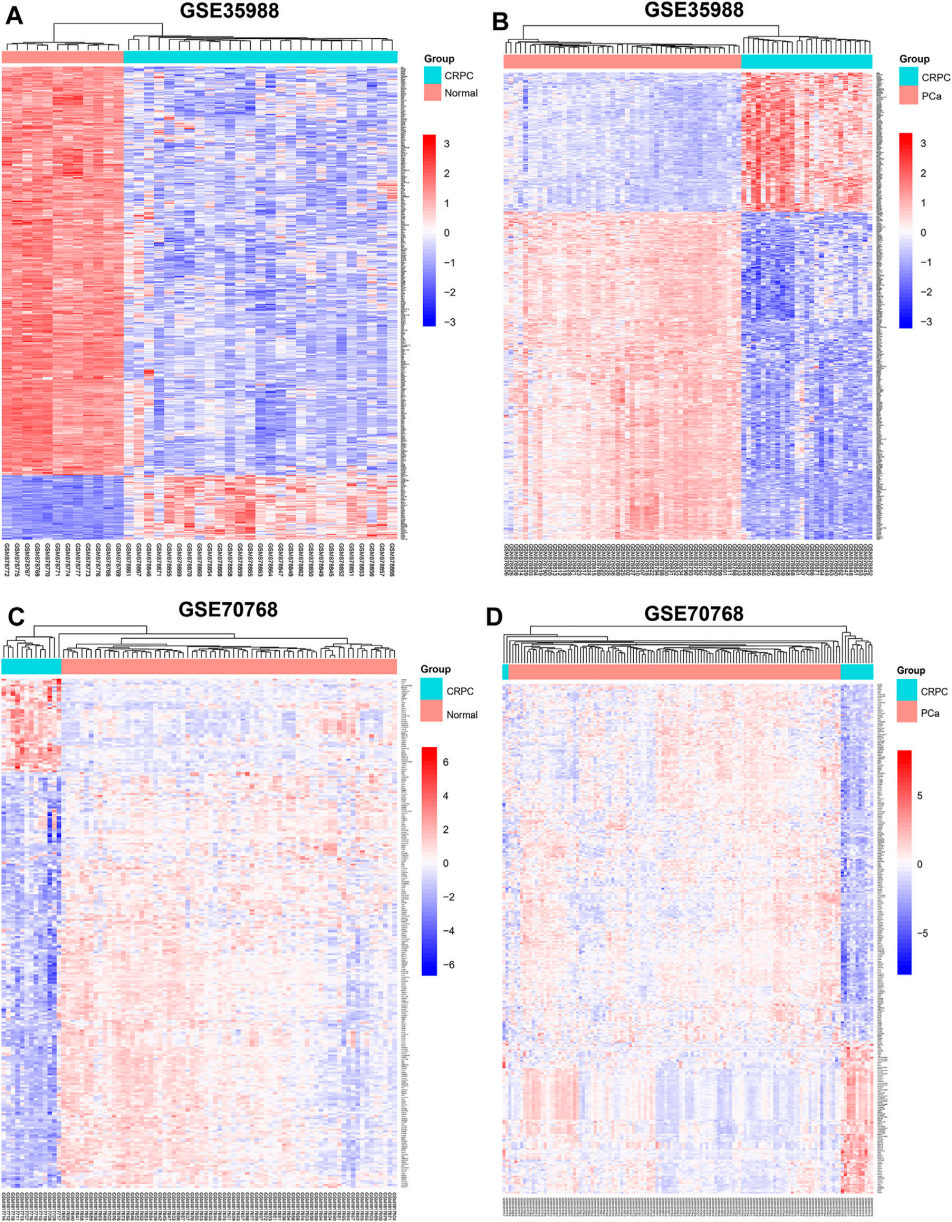
Heatmap of DEGs compared CRPC with normal and PCa samples. **(A,C)** DEGs of CRPC compared with normal samples in GSE35988 and GSE70768. **(B,D)** DEGs of CRPC compared with PCa samples in GSE35988 and GSE70768.

**FIGURE 2:**
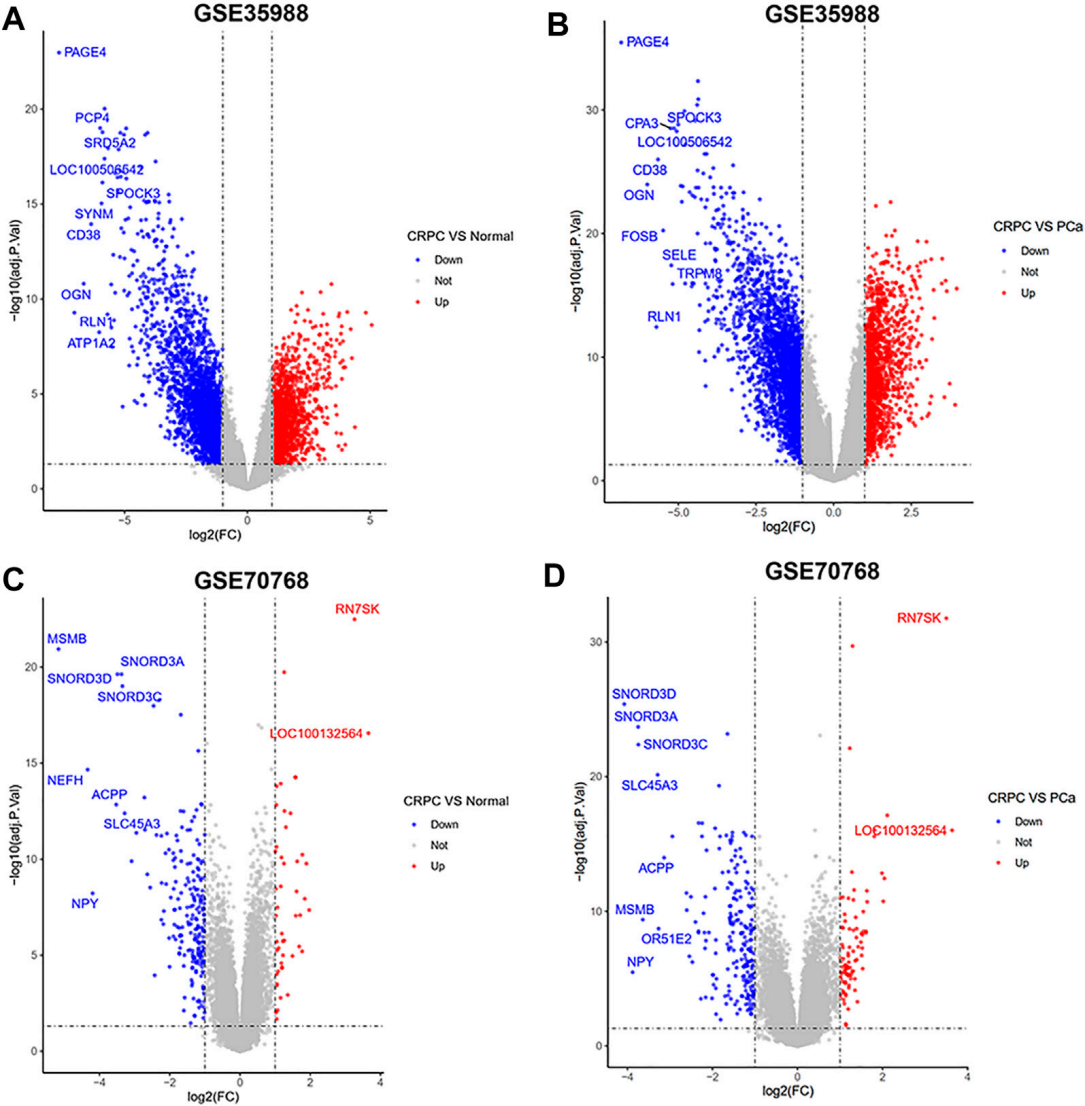
Volcano plots of DEGs compared CRPC with normal and PCa samples. **(A,C)** DEGs of CRPC compared with normal samples in GSE35988 and GSE70768. **(B,D)** DEGs of CRPC compared with PCa samples in GSE35988 and GSE70768.

**FIGURE 3:**
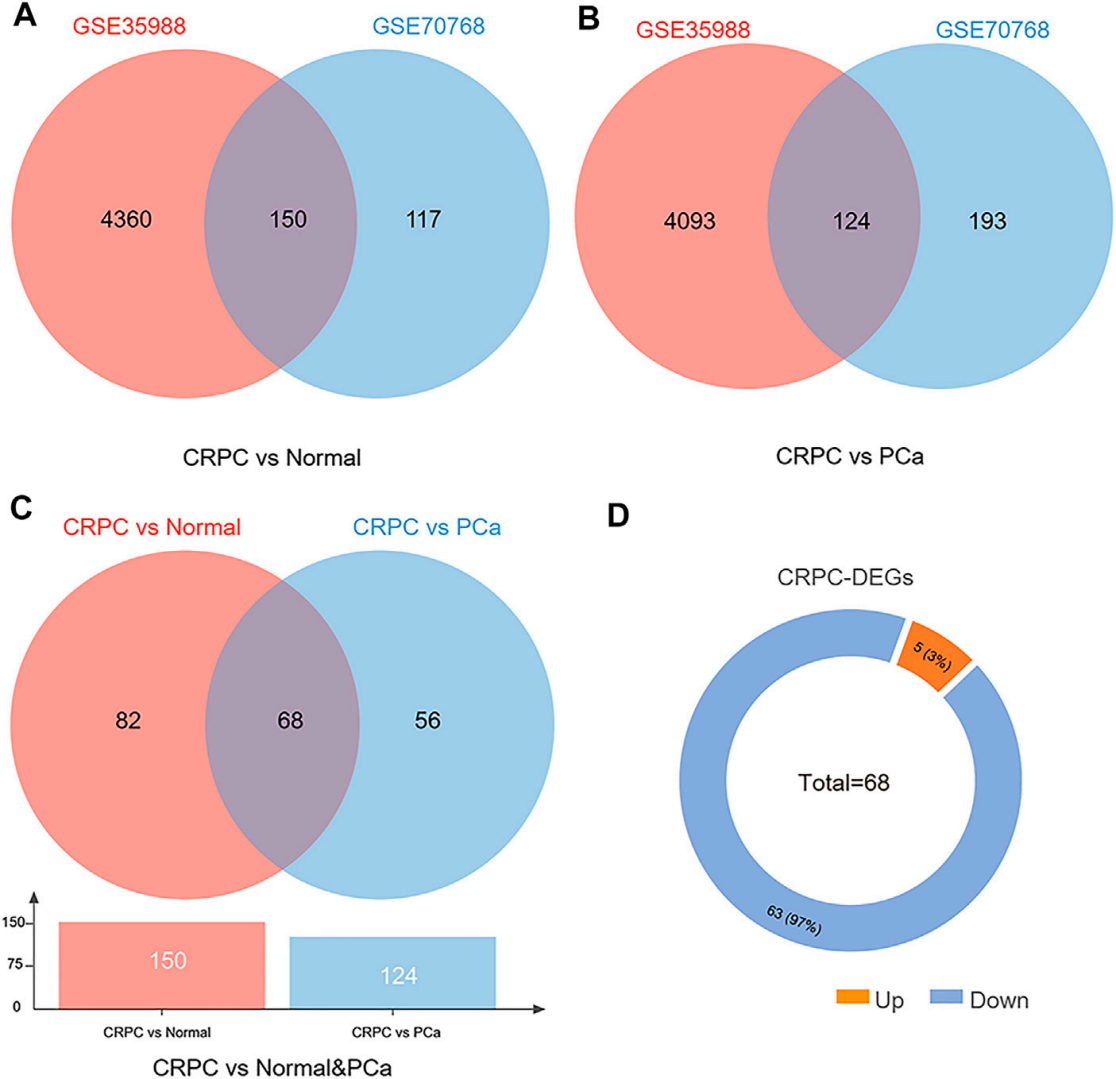
Venn diagram of DEGs compared CRPC with normal and PCa samples. **(A)** DEGs of CRPC compared with normal samples in both GSE35988 and GSE70768. **(B)** DEGs of CRPC compared with PCa in both GSE35988 and GSE70768. **(C)** DEGs of CRPC compared with normal and PCa samples in both GSE35988 and GSE70768. **(D)** The number of upregulated and downregulated DEGs.

The dataset of GSE35988–GPL6480 included 12 benign prostate tissue (normal) samples, 49 PCa samples, and 27 CRPC samples. The dataset of GSE70768–GPL10558 included 73 normal samples, 113 PC samples, and 13 CRPC samples. There were 4510 (1728 upregulated and 2782 downregulated genes) and 267 (49 upregulated and 218 downregulated genes) DEGs of CRPC compared with normal samples in GSE35988 and GSE70768, respectively (Figures 1A,C, 2A,C; Supplementary Table S1). At the same time, 4217 (1544 upregulated and 2673 downregulated genes) and 317 (95 upregulated and 222 downregulated genes) DEGs of CRPC compared with PCa samples were found in GSE35988 and GSE70768, respectively (Figures 1B,D, 2B,D; Supplementary Table S1). The top 10 DEGs with the largest change in the expression profile are shown in Figures 2A–D; Supplementary Table S2. Then, 150 DEGs distinguishing CRPC from normal samples and 124 DEGs distinguishing CRPC from PCa samples were obtained in both GSE35988 and GSE70768, respectively, by Venn analysis (Figures 3A,B). Finally, we got 68 CRPC-DEGs, including five upregulated and 63 downregulated genes, distinguishing CRPC from both normal and PC samples (Figures 3C,D; Supplementary Table S3).

### Genes in CRPC-Related Modules Identified by WGCNA

**FIGURE 4:**
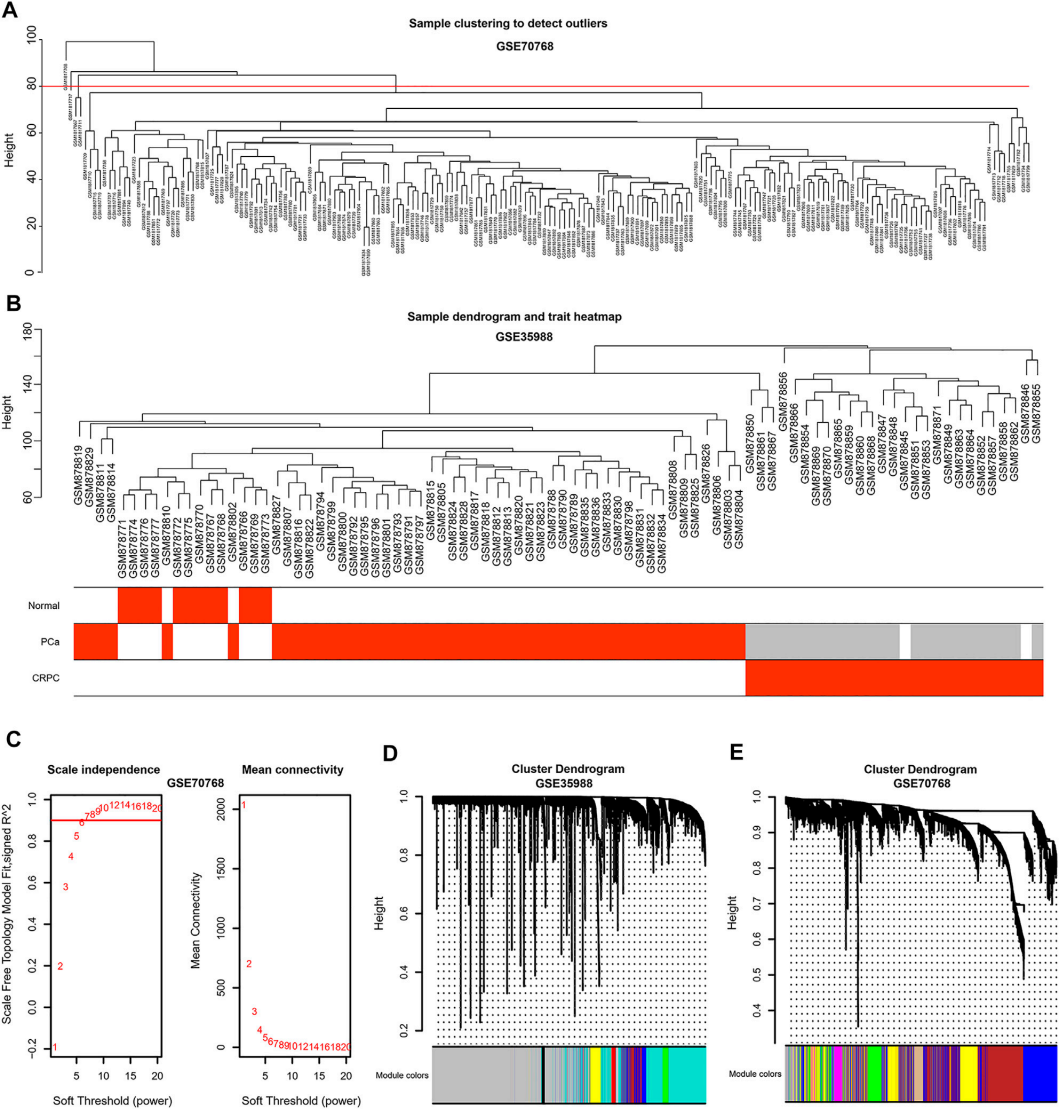
Sample clustering by WGCNA **(A)** Detection of the outliers by sample clustering in GSE70768. **(B)** The sample dendrogram with trait heatmap in GSE35988 **(C)** Identification of the optimal soft threshold power (β) in GSE70768. **(D,E)** The gene clustering dendrogram and the merged modules in GSE35988 and GSE70768.

**FIGURE 5:**
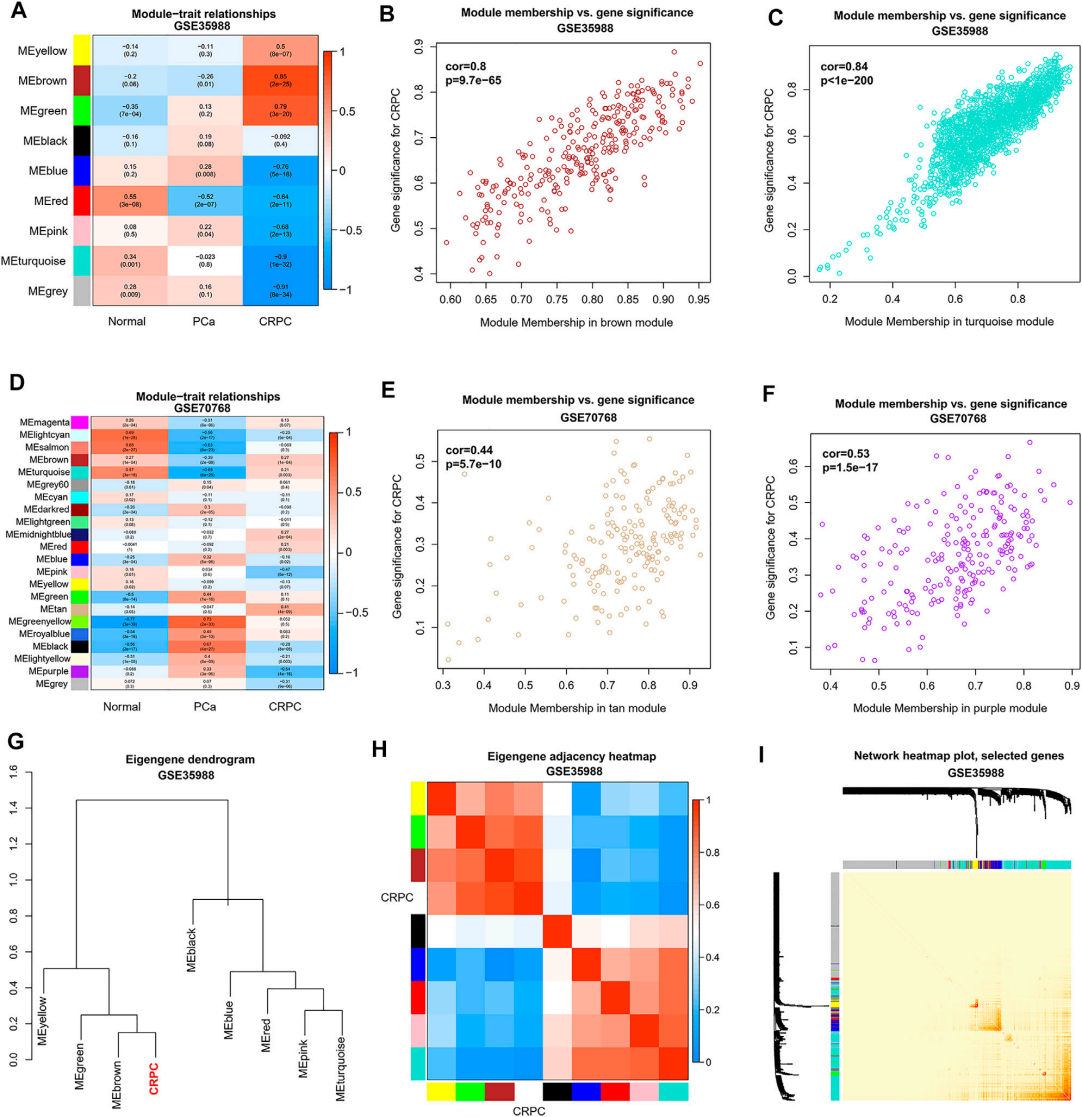
Identification of CRPC-related modules and genes by WGCNA. **(A,D)** Heatmap of correlation between modules and sample types. **(B,C,E,F)** Relationship between genes and traits in the CRPC-related modules. **(G,H)** Correlation between CRPC and different modules in dendrogram and heatmap. **(I)** Heatmap of a weighted network using 500 randomly selected genes.

To obtain the genes in CRPC-related modules, WGCNA was performed in each dataset of GSE35988 and GSE70768, respectively. Four samples in GSE70768 were detected as outliers by sample clustering (Figure 4A). Sample dendrogram and its relationship with clinical traits are also displayed in Figure 4B; Supplementary Figure S2. TOM was constructed when the optimal soft threshold power (β) was equal to 14 and six in each of GSE35988 and GSE70768, respectively (Figure 4C; Supplementary Figure S3). Then, all genes of normal, PCa, and CRPC samples were divided into different modules by dynamic tree cutting (Figures 4D,E). From the heatmap of module–trait relationships, we obtained 199 genes in the brown module and 171 genes in the tan module positively correlated with CRPC, as well as 1213 genes in the turquoise module and 186 genes in the purple module negatively correlated with CRPC (Figures 5A–H). The TOM plot of 500 randomly selected genes was also performed to visualize the network connections (Figure 5I).

### Functional Enrichment Analysis

**FIGURE 6:**
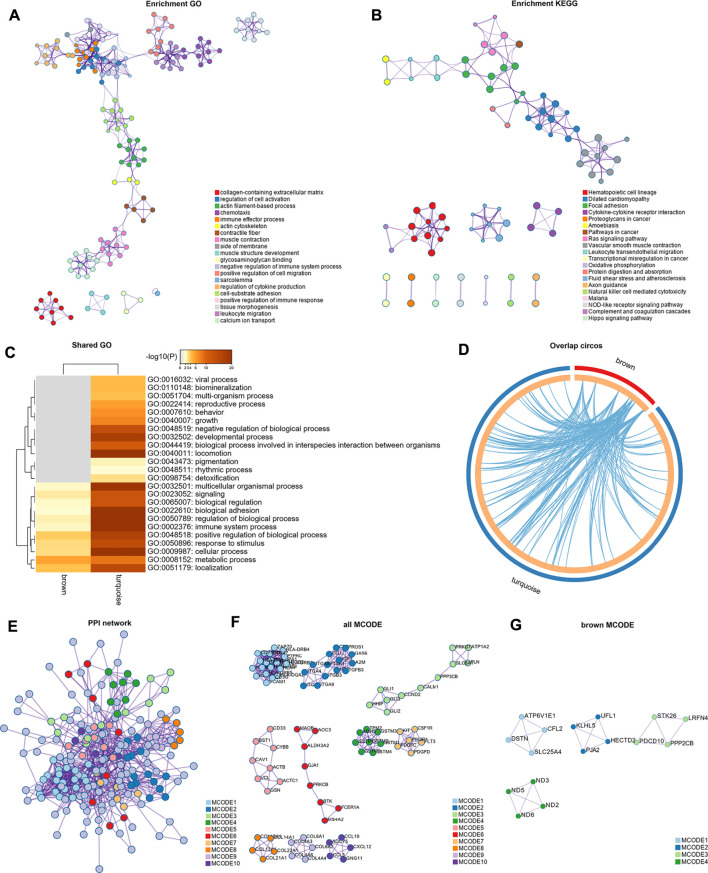
Functional enrichment analysis and construction of PPI network in the brown and turquoise modules. **(A)** GO terms of CRPC-related genes. **(B)** KEGG pathways of CRPC-related genes. **(C)** GO terms shared by both brown and turquoise modules. **(D)** Genes in different modules shared by the same GO terms. **(E)** PPI network of all proteins in the brown and turquoise modules. **(F)** Functional modules in the whole PPI network. **(G)** Functional modules in the brown modules.

**FIGURE 7:**
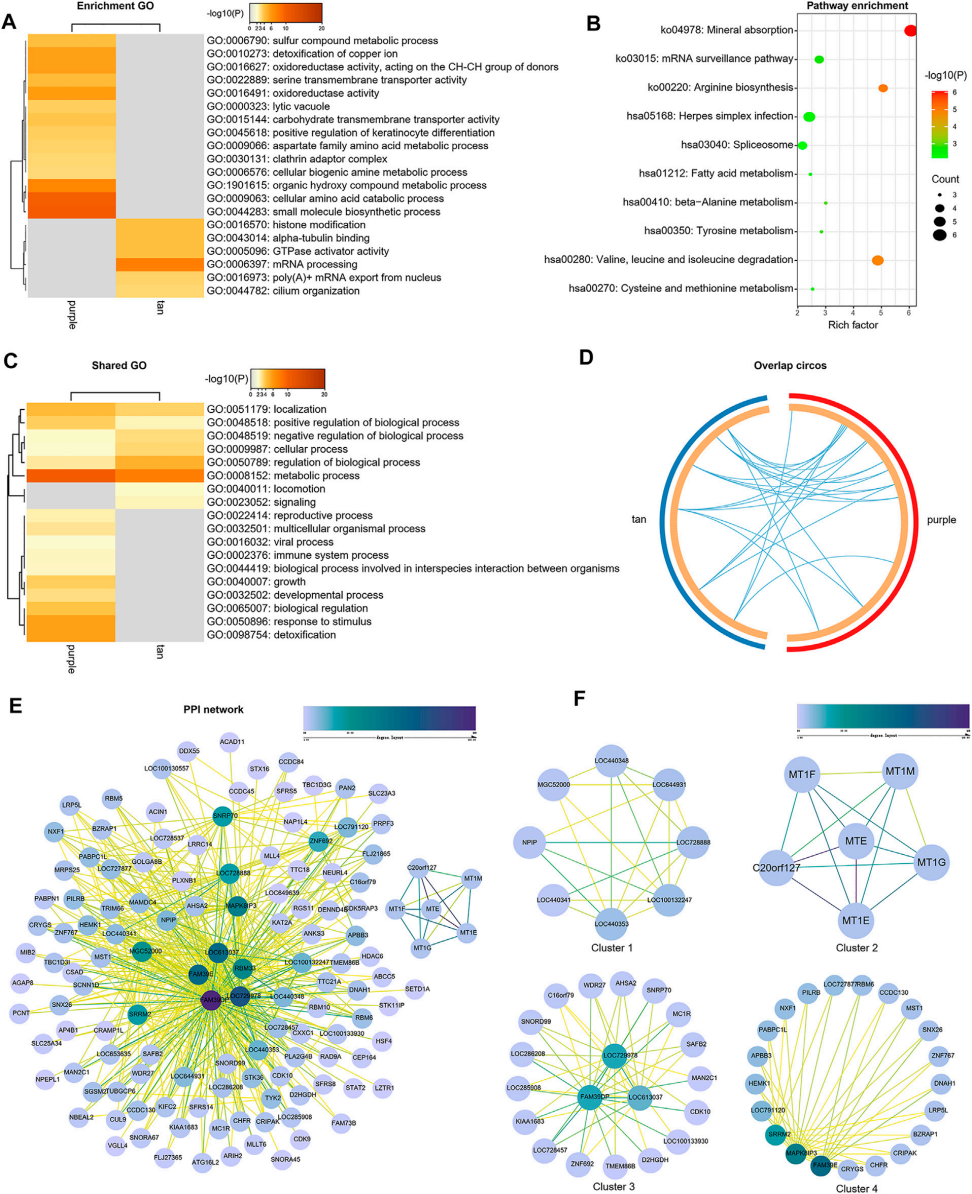
Functional enrichment analysis and construction of PPI network in the tan and purple modules. **(A)** GO terms of CRPC-related genes. **(B)** KEGG pathways of CRPC-related genes. **(C)** GO terms shared by both tan and purple modules. **(D)** Genes in different modules are shared by the same GO terms. **(E)** PPI network of all proteins in the tan and purple modules. **(F)** Functional modules in the whole PPI network.

Genes of GSE35988 in brown and turquoise modules as well as the genes of GSE70768 in tan and purple modules were used for performing GO and KEGG analysis (Supplementary Table S4). The top 20 enriched terms of GO and KEGG with a similarity >0.3 were rendered as a network (Figures 6A,B, 7A,B). The shared GO terms mainly included metabolic process, regulation of the biological process, cellular process, and localization (Figures 6C, 7C). Genes in different modules shared by the same GO terms are shown in Figures 6D, 7D. The Molecular Complex Detection (MCODE) networks identified by the PPI analysis are shown in Figures 6E–G–G, 7E,F.

### Development of a Two-Gene Signature for CRPC

**FIGURE 8:**
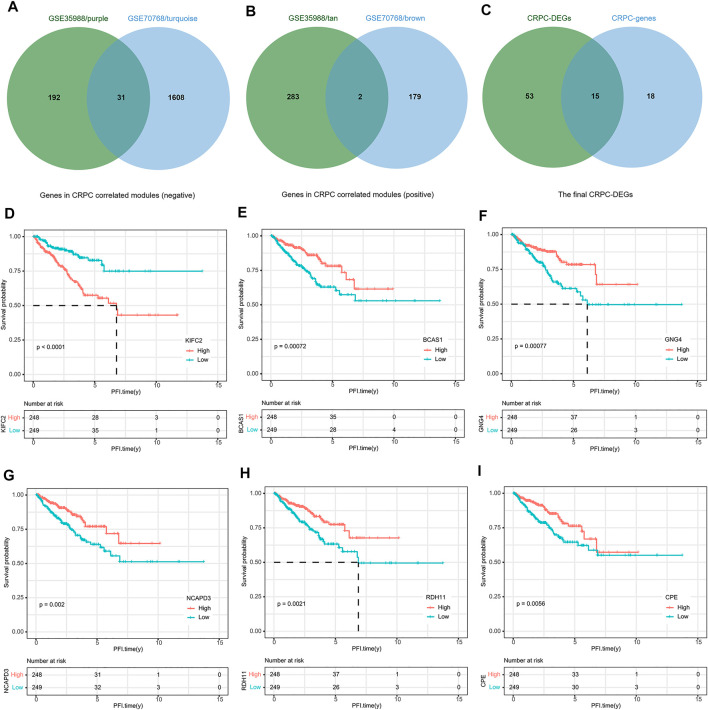
Identification of DEGs associated with PFI. **(A)** Venn diagram of genes negatively correlated with CRPC. **(B)** Venn diagram of genes positively correlated with CRPC. **(C)** Identification of the final 15 DEGs. **(D–I)** Representative DEGs significantly associated with PFI.

**FIGURE 9:**
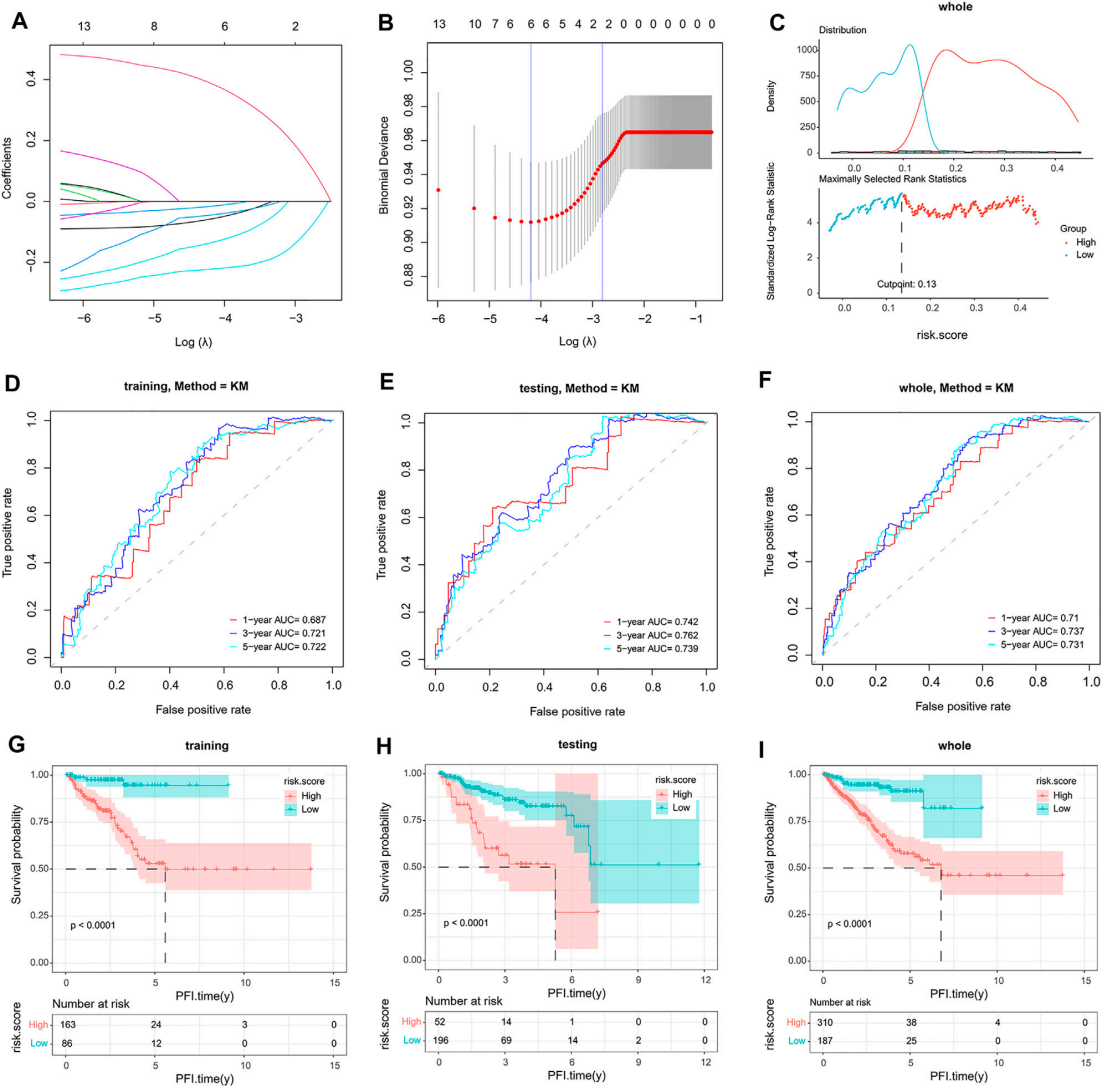
Construction and validation of a two-gene signature. **(A,B)** Identification of two genes and their coefficients in the optimal prognostic model by lasso-penalized Cox regression analysis. **(C)** Determination of the optimal cutoff value in the whole dataset. **(D–F)** The predictive capacity of the model verified by the ROC analysis in the training, testing, and whole dataset. **(G–I)**. Kaplan–Meier survival analysis of patients with a high-risk and low-risk score in the training, testing, and whole dataset.

To build this CRPC prognostic model, 497 PCa patients from the TCGA dataset were included for further analysis. Then, 15 final CRPC-DEGs were obtained by Venn analysis (Figures 8A–C). The enrichment analysis results of the 15 CRPC-DEGs are provided in Supplementary Table S5, of which 13 CRPC-DEGs statistically associated with PFI were identified as the candidate genes by Kaplan–Meier survival analysis (Figures 8D–I; Supplementary Figure S4). Next, the lasso-penalized Cox regression analysis was used to screen out the ideal prognostic genes by compressing the insignificant variable coefficients to 0. The trajectory of 13 candidate genes is shown in Figure 9A. When the lambda was equal to 0.06030151 (Log(λ) = −2.808398), the optimal prognostic model was obtained (Figure 9B), and two genes (KIFC2 and BCAS1) were selected finally to build the model (Supplementary Table S6). The risk score of each patient was calculated according to the following formula: risk score = 0.12466577 × Exp (KIFC2)-0.09149851 × Exp (BCAS1). The whole dataset was then separated into a training dataset (*n* = 249) and a testing dataset (*n* = 248) randomly. Depending on the optimal cutoff value determined by the “surv_cutpoint” function, the patients in each of the training, testing, and whole dataset were separated into high-risk and low-risk groups (Figure 9C; Supplementary Figure S5). The predictive capacity of the two-gene based model for CRPC was assessed by the ROC curve and Kaplan–Meier survival curve in each dataset (Figures 9D–I). As a representative, the 1-year, 3-year, and 5-year area under curve (AUC) for PFI in the whole dataset were 0.71, 0.737, and 0.731, respectively (Figure 9F). Moreover, patients in the training, testing, and whole dataset with high-risk scores were significantly associated with poorer PFI (*p* < 0.0001; Figures 9G–I).

### Expression Profiles of the Two Genes in the Model

**FIGURE 10:**
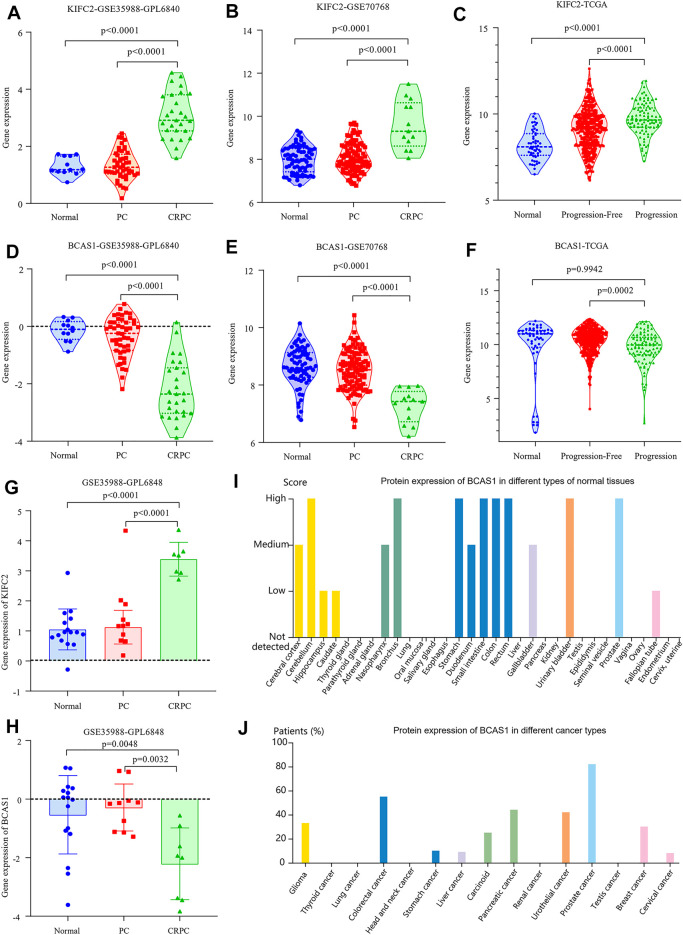
Expression of KIFC2 and BCAS1. **(A,B,G)** The gene expression of KIFC2 is higher in CRPC than in normal and PCa samples. **(D,E,H)** The gene expression of BCAS1 is lower in CRPC than in normal and PCa samples. **(C,F)** While the gene expression of KIFC2 is higher in patients with progression, that of BCAS1 is lower in patients with progression. **(I,J)** The protein expression of BCAS1 is high in many normal tissues including prostate but low in most cancer types excluding prostate cancer.

To further understand the relationship between the two genes (KIFC2 and BCAS1) and CRPC, we analyzed their expression levels in the dataset of GSE35988, GSE70768, and TCGA, respectively. The expression of KIFC2 was not only higher in the CRPC samples than that in normal and PCa samples but also highly expressed in the progression group compared with the normal and progression-free groups (*p* < 0.0001, Figures 10A–C,G). For BCAS1, it was expressed lower in the CRPC samples than in normal and PCa samples (*p* < 0.0001, Figures 10D,E,H). Although the expression of BCAS1 in the progression group was lower than that in the progression-free group, there was no significant difference between the progression and normal groups (Figure 10F). Based on the HPA database, it was found that the expression of KIFC2 was low in prostate and prostate cancer tissue with no mention of CRPC, as well as low and medium expression in a variety of normal tissues and cancer types (Supplementary Figure S6). Although BCAS1 was highly expressed in a variety of normal tissues including the prostate, it was lowly expressed in many different cancer types except prostate cancer (Figures 10I,J).

### Establishment of a Combined Nomogram for Individualized Prediction

**FIGURE 11:**
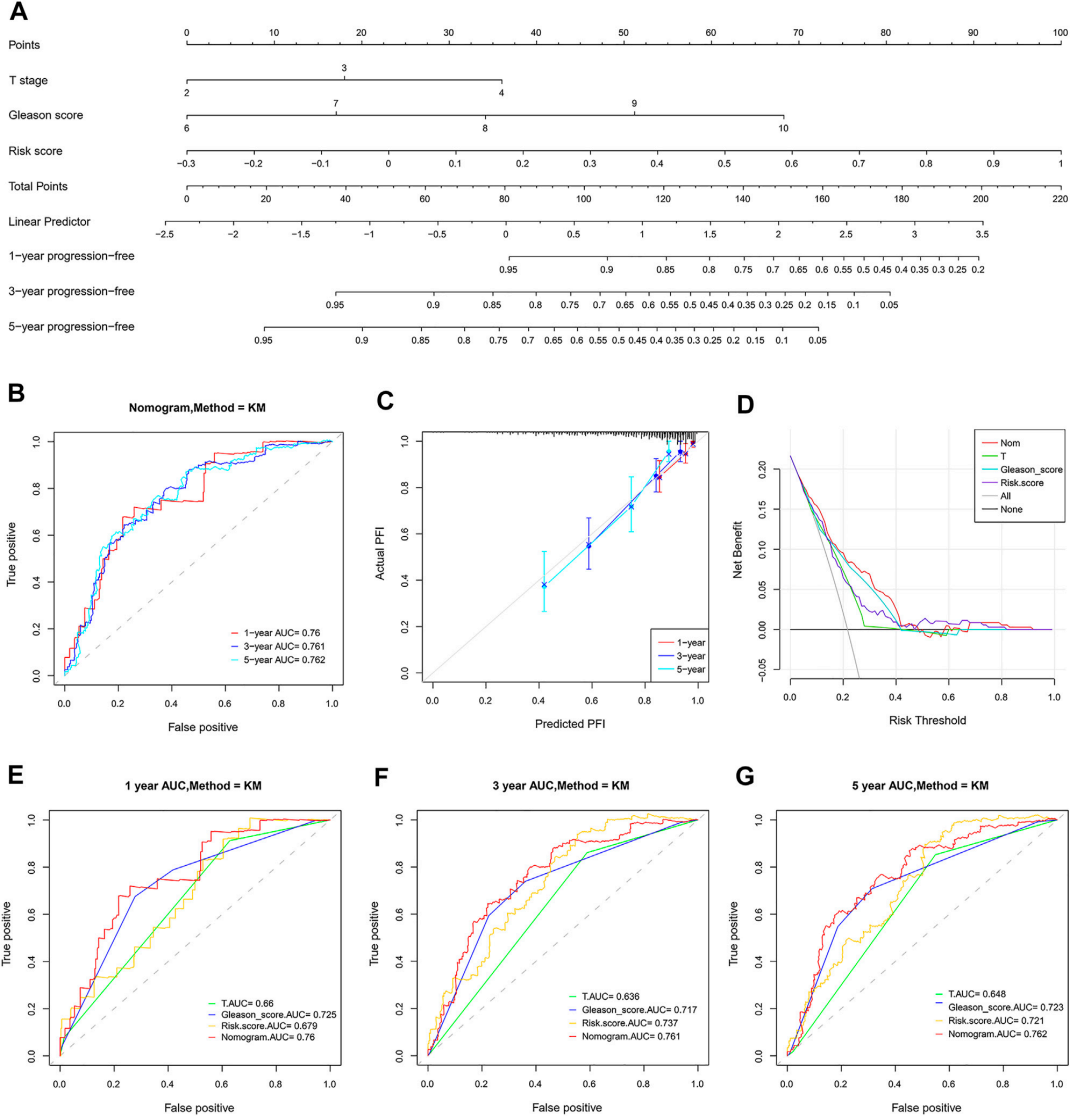
Establishment and validation of a combined nomogram. **(A)** The nomogram predicts PFI based on risk score, T stage, and Gleason score. **(B,E–G)** The prognostic value of the nomogram is better than that of any single factor confirmed by the ROC analysis. **(C)** Predictive capacity of the nomogram assessed by the calibration plots. **(D)** Predictive capacity of the nomogram assessed by DCA.

The risk score and other clinical factors, including age, T stage, N stage, laterality, Gleason score, and PSA, were used for univariate and multivariate Cox regression analyses (Table 1). The results showed that the two-gene signature was independent of other clinical factors. By the multivariate Cox regression analysis, a nomogram integrated with the T stage, Gleason score, and risk score was established (Figure 11A). The C-index was 0.734, indicating a good consistency. As shown in the ROC curve, the 1-year, 3-year, and 5-year AUC of the nomogram were 0.76, 0.761, and 0.762, respectively, which were larger than those of any single factor (Figures 11B,E–G). The calibration plots and DCA curve also showed that the nomogram performed well in the individualized prediction of progression to CRPC (Figures 11C,D).

**TABLE 1:** Results of univariate and multivariate Cox regression analyses.

Characteristic	Univariate Cox regression		Multivariate Cox regression	
	Hazard ratio (95% CI)	*p*-value	Hazard ratio (95% CI)	*p*-value
Age	1.02 (0.9852–1.056)	0.265	9.960e-01 (0.9626–1.031)	0.820141
T	2.695 (1.711–4.247)	1.91e-05	1.805 (1.0345–3.148)	0.037576
N	1.595 (0.9474–2.686)	0.0789	7.504e-01 (0.4278–1.317)	0.316797
M	8.245e-07 (0-Inf)	0.995	2.727e-07 (0-Inf)	0.994918
Laterality	1.303 (0.5655–3.001)	0.535	9.159e-01 (0.3914–2.143)	0.839571
Gleason score	2.156 (1.695–2.741)	3.64e-10	1.661 (1.2499–2.206)	0.000466
PSA	1.063 (1.028–1.1)	0.000328	1.036 (0.9983–1.074)	0.061697
Risk score	34.17 (10.57–110.4)	3.61e-09	1.031e+01 (2.9291–36.297)	0.000279

## Discussion

CRPC is a lethal stage for all PCa patients, which will continue to progress despite continuous androgen deprivation therapy (ADT) and maintaining a testosterone level of less than 50 ng/dl (Lokeshwar et al., 2021; Schaeffer et al., 2021). Even if many new therapeutic options have emerged in recent years, CRPC remains incurable at present and for a long time in the future (Mateo et al., 2019; Nuhn et al., 2019; De Porras et al., 2021; Lowrance et al., 2021). Many studies have confirmed that in-depth mining of tumor-related transcriptome data can help us further understand the biological characteristics of tumors, so as to improve their clinical prognosis and therapeutic effect (Chen et al., 2019; Lawson et al., 2020; Chakravarty and Solit, 2021; Piccart et al., 2021). Given the current limited monitoring and treatment capacities, we developed a novel combined nomogram for predicting the individualized risk of progression related to castration resistance.

To obtain the DEGs distinguishing CRPC from normal and PCa samples, we first compared the gene expression level of CRPC with that of normal and PCa, and then performed the WGCNA. The brown, turquoise, tan, and purple modules associated with CRPC were identified. Enrichment analysis was conducted to further explore the function of genes in the four modules. The shared GO showed that the main biological processes included localization, metabolic process, cellular process, and regulation of the biological process, which were likely related to the progression and drug resistance of CRPC. Using the Venn and Kaplan–Meier survival analyses, 13 CRPC-DEGs significantly associated with PFI were finally identified as the candidate genes for modeling.

Based on the results of lasso-penalized Cox regression analysis, a two-gene signature was built by the gene expression level and regression coefficient of KIFC2 and BCAS1. According to the risk score, patients in the TCGA dataset were then divided into high-risk and low-risk groups. The Kaplan–Meier survival curve showed that patients with high-risk scores had a significantly poorer PFI than those with low-risk scores, suggesting that patients with high-risk scores were more prone to progression. Furthermore, the prediction ability of the model has been fully confirmed by the ROC curve in different datasets. Therefore, the two-gene signature prognostic model can effectively predict the risk of castration resistance in PCa patients.

The nomogram has been widely used by oncologists to generate prognostic information for the individual patient due to its numerical probability and user-friendly interface (Iasonos et al., 2008; Gandaglia et al., 2020; Diamand et al., 2021). In this study, a novel nomogram was established by integrating the risk score, T stage, and Gleason score, each of which was an independent prognostic factor in the light of multivariate Cox regression analysis. As a comprehensive scoring system, the combined nomogram is better than any single factor in risk prediction and patient stratification, which was finally confirmed by the results of the C-index, calibration plots, ROC, and DCA curve. In clinical practice, when a patient with prostate cancer comes to a doctor, the T stage can be determined by magnetic resonance imaging, and the Gleason score and risk score can be determined by prostate biopsy. The total points of all variables in the nomogram can be used to predict the disease progression of prostate cancer, especially the risk of CRPC-related progression, so as to take appropriate treatment measures or adjust the original treatment plan in time.

In terms of genes in the model, KIFC2 encodes a kinesin-like protein consisting of 792 amino acids and has ATP-dependent microtubule motor activity (Hirokawa et al., 1998). The previous study suggested that KIFC2 was mainly expressed in adult neurons and associated with the multivesicular body (mvb)-like organelles (Saito et al., 1997). Recently, it was found that downregulated KIFC2 would lead to a significant decrease in the number of neuronal dendrites, indicating that KIFC2 is critical for dendrite development (Szczurkowska et al., 2020). Furthermore, KIFC2 was reported as one of the 19 markers in a panel of DNA methylation for the detection of prostate cancer from FV and DRE urine DNA (Brikun et al., 2018). So far, no report about the relationship between KIFC2 and tumor progression has been found. In this study, we found that the gene expression level of KIFC2 in CRPC samples was significantly higher than that in normal and PCa samples for the first time. In addition, patients with higher expression of KIFC2 had poorer PFI, suggesting that the increased expression of KIFC2 is associated with the increased risk of progression related to castration resistance in PCa patients.

BCAS1 is a protein-encoding gene located at 20q13.2. Since the BCAS1 gene was originally found in human breast carcinoma cells, its previous name was “breast carcinoma amplified sequence 1” (Collins et al., 1998). Recent studies have found that BCAS1 is highly expressed in the brain, especially in oligodendrocytes, so its name is now approved as “brain-enriched myelin-associated protein 1” (Fard et al., 2017). For cancer patients, the expression level of BCAS1 affects prognosis variously among different tumor types (McFarlane et al., 2018; Zhang et al., 2019). Even BCAS1 is associated with drug resistance in cancer patients during treatment (Jing et al., 2020). More importantly, BCAS1 can be used as an accurate marker in the urine cell-free DNA analysis for early prostate cancer diagnosis (Casadio et al., 2013). In our results, the expression of BCAS1 was low in CRPC samples and significantly influenced the PFI, indicating that patients with high expression of BCAS1 are less likely to experience the transformation from PCa to CRPC.

## Conclusion

In summary, genes in the CRPC-related modules were mainly involved in the metabolism, localization, and regulation of the biological process. In addition to the DEGs distinguishing CRPC from both normal prostate and PCa, a two-gene signature was developed based on KIFC2 and BCAS1 in this study, and could be used as a marker for predicting the progression related to castration resistance in PCa patients. In addition, a novel combined nomogram was established and validated for individualized risk prediction of progress to CRPC. Although the prognostic ability of this nomogram has been preliminarily confirmed, it still needs further demonstration by clinical research, which will be our effort in the future.

## Data Availability

Publicly available datasets were analyzed in this study. These data can be found here: https://www.ncbi.nlm.nih.gov/geo/query/acc.cgi?acc=GSE35988, https://www.ncbi.nlm.nih.gov/geo/query/acc.cgi?acc=GSE70768, https://xenabrowser.net/datapages/?cohort=TCGA%20Prostate%20Cancer%20(PRAD)& removeHub=https%3A%2F%2Fxena.treehouse.gi.ucsc.edu%3A443.
