# Supplementary figures and images for: Establishment of a Novel Combined Nomogram for Predicting the Risk of Progression Related to Castration Resistance in Patients With Prostate Cancer
Source: Front Genet. 2022 May 10;13:823716. doi: 10.3389/fgene.2022.823716 (PMC9127235; doi:10.3389/fgene.2022.823716)

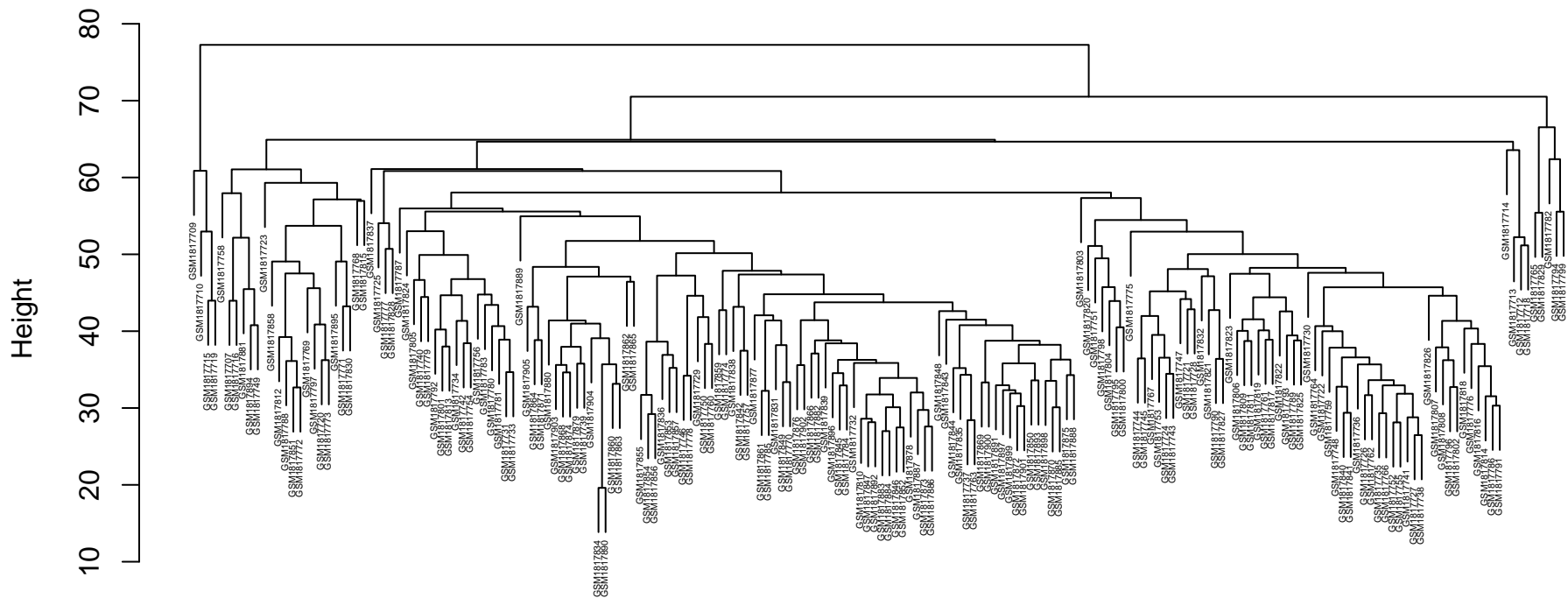

Supplement: Supplementary file 1 [file DataSheet2.PDF]

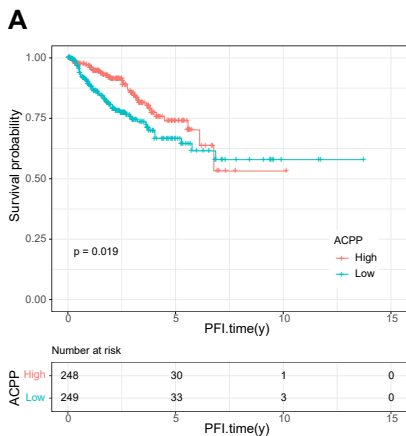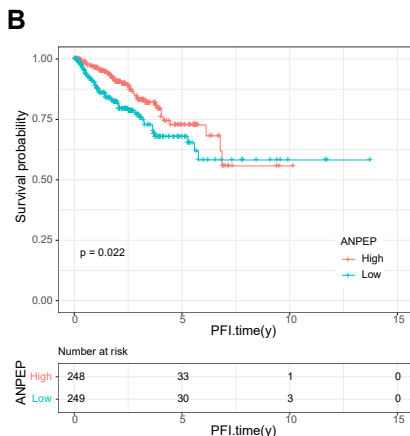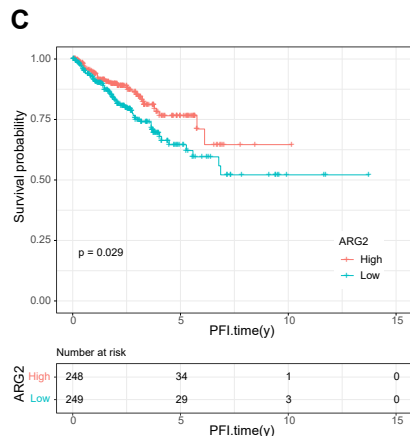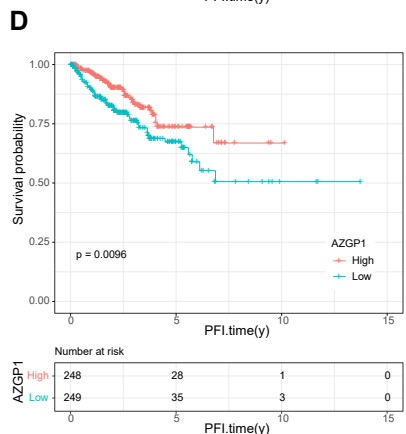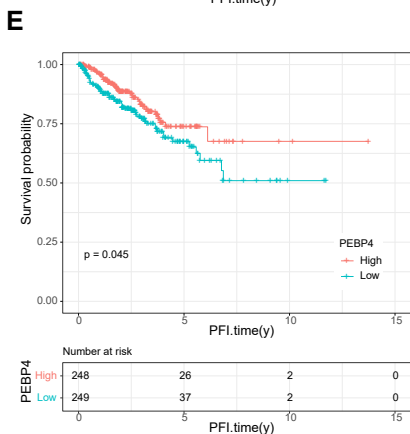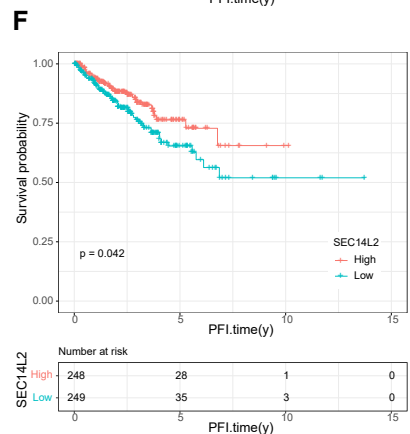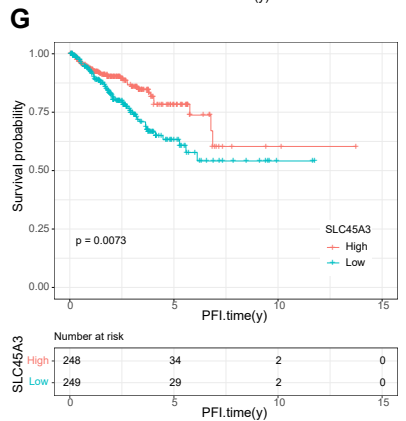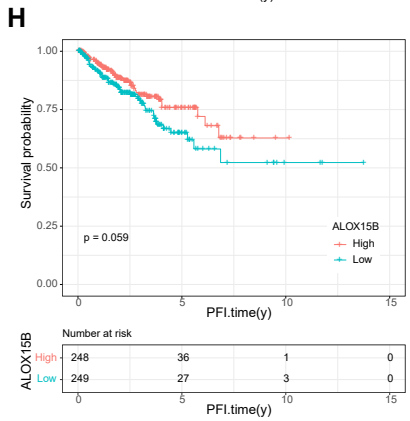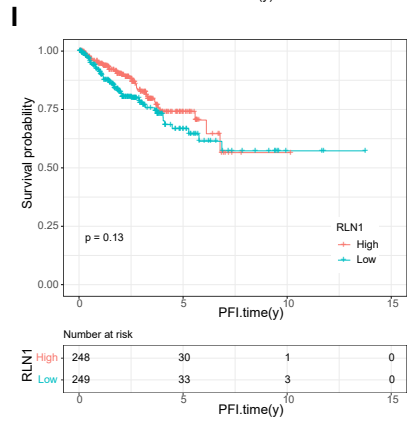

Supplement: Supplementary file 2 [file DataSheet4.PDF]

# Scale independence

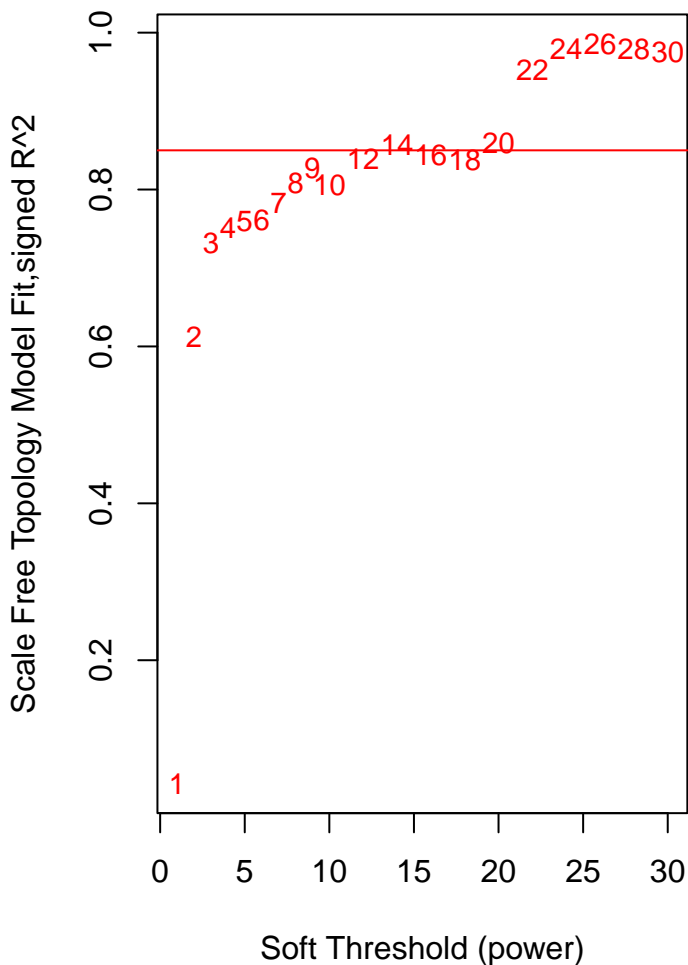

# Mean connectivity

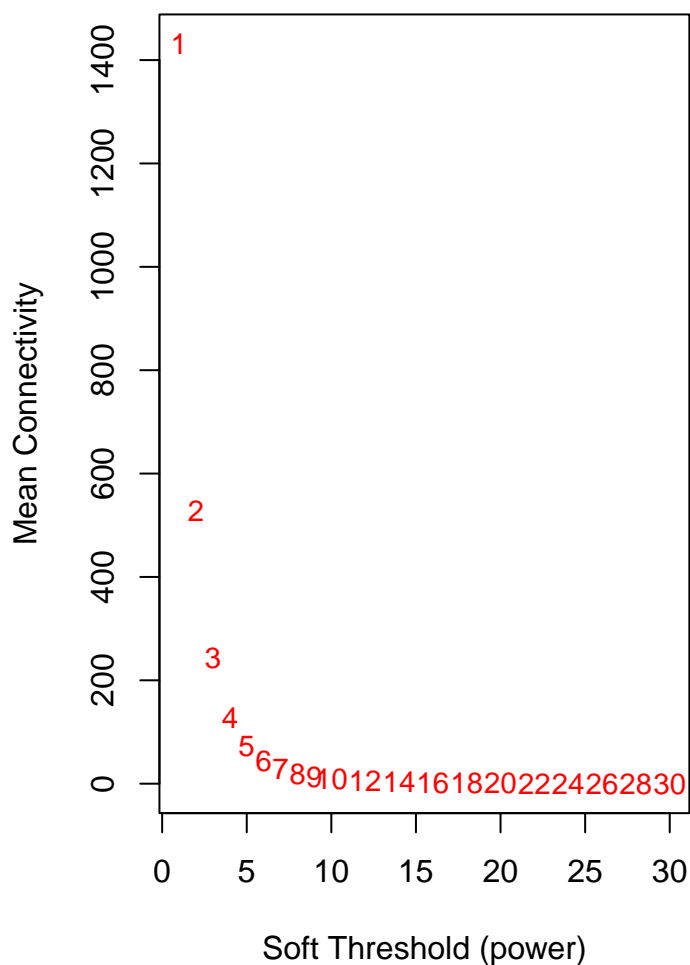

Supplement: Supplementary file 4 [file DataSheet3.PDF]

- GEO
- Datasets
- Samples:  
CRPC  
PCa  
Normal

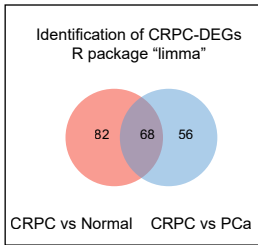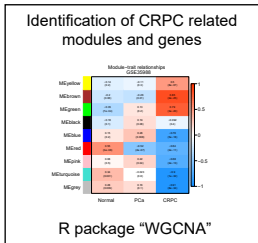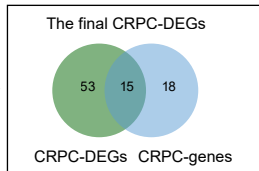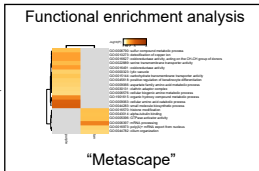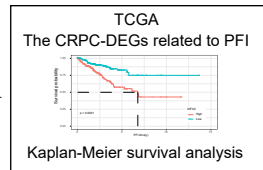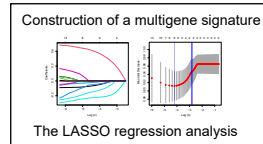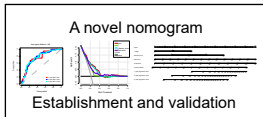

Supplement: Supplementary file 6 [file DataSheet1.PDF]

**A**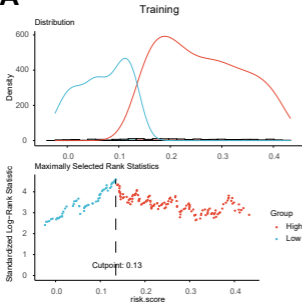**B**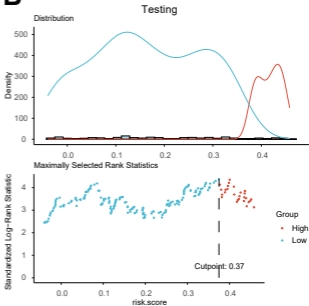

Supplement: Supplementary file 7 [file DataSheet5.PDF]
