# Supplementary material for: Establishment of a Novel Combined Nomogram for Predicting the Risk of Progression Related to Castration Resistance in Patients With Prostate Cancer
Source: Front Genet. 2022 May 10;13:823716. doi: 10.3389/fgene.2022.823716 (PMC9127235; doi:10.3389/fgene.2022.823716)
Supplement: Supplementary file 3 [file DataSheet6.PDF]

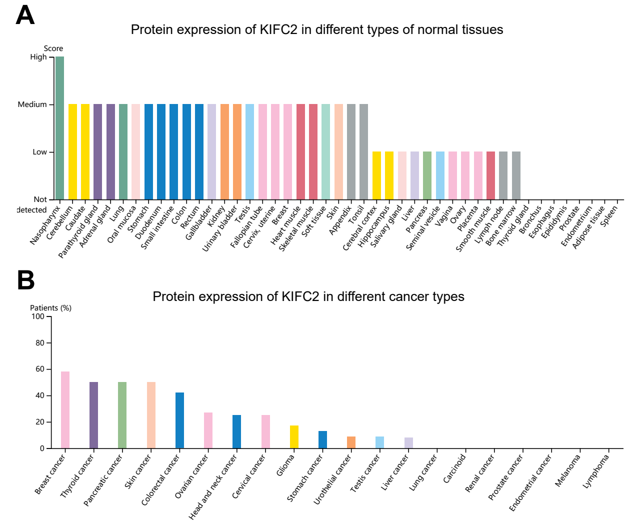
A
Protein expression of KIFC2 in different types of normal tissues
Score
High
Medium
Low
Not detected
Nasopharynx
Cerebellum
Caudate
Parathyroid gland
Adrenal gland
Lung
Oral mucosa
Stomach
Duodenum
Small intestine
Colon
Rectum
Gallbladder
Kidney
Urinary bladder
Testis
Fallopian tube
Cervix, uterine
Breast
Heart muscle
Skeletal muscle
Soft tissue
Skin
Appendix
Tonsil
Cerebral cortex
Hippocampus
Salivary gland
Liver
Pancreas
Seminal vesicle
Vagina
Ovary
Placenta
Smooth muscle
Lymph node
Bone marrow
Thyroid gland
Bronchus
Esophagus
Epididymis
Prostate
Endometrium
Adipose tissue
Spleen
B
Protein expression of KIFC2 in different cancer types
Patients (%)
100
80
60
40
20
0
Breast cancer
Thyroid cancer
Pancreatic cancer
Skin cancer
Colorectal cancer
Ovarian cancer
Head and neck cancer
Cervical cancer
Glioma
Stomach cancer
Urothelial cancer
Testis cancer
Liver cancer
Lung cancer
Carcinoid
Renal cancer
Prostate cancer
Endometrial cancer
Melanoma
Lymphoma
