# Supplementary material for: Establishment of a Novel Combined Nomogram for Predicting the Risk of Progression Related to Castration Resistance in Patients With Prostate Cancer
Source: Front Genet. 2022 May 10;13:823716. doi: 10.3389/fgene.2022.823716 (PMC9127235; doi:10.3389/fgene.2022.823716)
Supplement: Supplementary file 5 [file Table2.DOCX]

**Table S1.** The top 10 DEGs with the largest change in expression profile

| CRPC vs Normal | |  | CRPC vs PCa | |
| --- | --- | --- | --- | --- |
| GSE35988 | GSE70768 |  | GSE35988 | GSE70768 |
| PAGE4  RLN1  OGN  CD38  ATP1A2  PCP4  SYNM  SPOCK3  SRD5A2  LOC100506542 | NTSR1  PBOV1  TAS2R50  MSMB  NEFH  NPY  LOC100132564  ACPP  SNORD3D  SNORD3A |  | PAGE4  OGN  RLN1  CD38  FOSB  CPA3  SELE  TRPM8  SPOCK3  LOC100506542 | SNORD3D  NPY  SNORD3A  SNORD3C  MSMB  LOC100132564  RN7SK  SLC45A3  OR51E2  ACPP |
