# Supplementary material for: Establishment of a Novel Combined Nomogram for Predicting the Risk of Progression Related to Castration Resistance in Patients With Prostate Cancer
Source: Front Genet. 2022 May 10;13:823716. doi: 10.3389/fgene.2022.823716 (PMC9127235; doi:10.3389/fgene.2022.823716)
Supplement: Supplementary file 9 [file Table3.DOCX]

**Supplement Table 2. The 68 DEGs of CRPC comparing with normal and PCa samples**

| CRPC vs Normal and PCa |
| --- |
| EMP1  KIFC2  NAAA  DBI  SOCS2  TSPAN8  ARG2  CECR6  CD9  ALDH1A3  KIAA1324  AZGP1  GNMT  CELSR3  MT1F  ZFP36  PEBP4  ACPP  PAK1IP1  SLC45A3  NCAPD3  GLB1L2  COX19  PAGE4  TMEM79  C1orf116  ALOX15B  TMSB15A  CUX2  GCAT  SEC11C  TRPM8  HERPUD1  RHOBTB3  RDH11  BCAS1  SORD  NANS  ANXA3  RASD1  COL1A1  MT1M  IDH1  TMPRSS2  ANPEP  CTGF  DUSP1  SC5DL  CREB3L4  EPHX2  KBTBD11  VSIG2  GNG4  H19  RLN1  UBE2J1  MESP1  CD38  XBP1  DHRS7  NEFH  CPE  MSMB  SEC14L2  NKX3-1  RAB3B  GMPR  SPOCK1 |
