# Supplementary material for: Establishment of a Novel Combined Nomogram for Predicting the Risk of Progression Related to Castration Resistance in Patients With Prostate Cancer
Source: Front Genet. 2022 May 10;13:823716. doi: 10.3389/fgene.2022.823716 (PMC9127235; doi:10.3389/fgene.2022.823716)
Supplement: Supplementary file 11 [file Table6.DOCX]

Table S4 The regression coefficients identified by lasso-penalized Cox regression analysis

| Gene | coefficient |
| --- | --- |
| ACPP | 0 |
| ANPEP | 0 |
| ARG2 | 0 |
| AZGP1 | 0 |
| BCAS1 | -0.09149851 |
| CPE | 0 |
| GNG4 | 0 |
| KIFC2 | 0.12466577 |
| NCAPD3 | 0 |
| RDH11 | 0 |
| SEC14L2 | 0 |
| SLC45A3 | 0 |
| PEBP4 | 0 |
